# FBLIM1 mRNA is a novel prognostic biomarker and is associated with immune infiltrates in glioma

**DOI:** 10.1515/med-2023-0863

**Published:** 2023-12-16

**Authors:** Yifan Deng, Kailiang Zeng, Diancheng Wu, Yunzhi Ling, Yu Tian, Yi Zheng, Shumin Fang, Xiaocong Jiang, Gang Zhu, Yanyang Tu

**Affiliations:** Department of Neurosurgery, The Huizhou Central People’s Hospital, Guangdong Medical University, Huizhou, Guangdong, China; Research Center, The Huizhou Central People’s Hospital, Guangdong Medical University, Huizhou, Guangdong, China; Department of Radiotherapy, The Huizhou Central People’s Hospital, Guangdong Medical University, Huizhou, Guangdong, China

**Keywords:** FBLIM1 mRNA, immune cell infiltration, prognosis, biomarker, bioinformatics analysis, glioma

## Abstract

Glioma is the most common primary brain tumor. Filamin-binding LIM protein 1 (FBLIM1) has been identified in multiple cancers and is suspected of playing a part in the development of tumors. However, the potential function of FBLIM1 mRNA in glioma has not been investigated. In this study, the clinical information and transcriptome data of glioma patients were, respectively, retrieved from the TCGA and CGGA databases. The expression level of FBLIM1 mRNA was shown to be aberrant in a wide variety of malignancies. Significantly, when glioma samples were compared to normal brain samples, FBLIM1 expression was shown to be significantly elevated in the former. A poor prognosis was related to high FBLIM1 expression, which was linked to more advanced clinical stages. Notably, multivariate analyses demonstrated that FBLIM1 expression was an independent predictor for the overall survival of glioma patients. Immune infiltration analysis disclosed that FBLIM1 expression had relevance with many immune cells. The results of RT-PCR suggested that FBLIM1 expression was markedly elevated in glioma specimens. Functional experiments unveiled that the knockdown of FBLIM1 mRNA suppressed glioma cell proliferation. In general, we initially discovered that FBLIM1 mRNA might be a possible prognostic marker in glioma.

## 1 Introduction

Primary central nervous system cancers account for approximately 1.6% of cancers diagnosed annually around the world, with gliomas being the most frequent histological type [1]. Gliomas are primary brain tumors that can originate from either glial or neuronal progenitor cells. The risk of developing gliomas increases with age, with the highest rates occurring in people over the age of 75 [2]. Glioblastomas are responsible for around 70–75% of all gliomas, whereas low-grade gliomas (LGGs) are responsible for approximately 20–25% of all gliomas [3,4]. Despite the discovery of some prognostic indicators including lncRNA FOXD1-AS1 and hemodynamic change, the prognosis for patients who have a high-grade glioma is often less favorable than that of patients with other types of gliomas [5]. Patients diagnosed with LGG have a survival time range of 1–15 years, but those diagnosed with glioblastoma have a survival time median of just 16 months [6]. It has been established that glioblastoma has the potential to be very aggressive. Although significant headway has been achieved in the development of innovative therapies for the treatment of cancer, very few treatments have been authorized for the treatment of LGG, and the outlook for LGG patients continues to be dismal [7,8]. Furthermore, as glioblastoma typically remains asymptomatic in its early stages, the majority of individuals are not diagnosed until they reach advanced disease stages or develop distant metastases [9,10]. Consequently, the discovery of new molecular markers and prognostic indicators for glioma is very necessary in order to improve therapy results and reduce the burden of illness.

Filamin binding LIM protein 1 (FBLIM1) was considered an essential component of cell-extracellular matrix adhesions. It has been discovered that filamins, as cytoplasmic proteins cross-linking actin filaments can coordinate the interaction between the cytoskeleton and the epidermal growth factor receptor (EGFR) [11]. Meanwhile, FBLIM1 is associated with inflammation-associated diseases, such as chronic recurrent multifocal osteomyelitis, and exists in numerous forms of malignancies, involving the brain, breast, liver, and other sites [12,13,14]. Moreover, chronic recurrent multifocal osteomyelitis is among the conditions that can be attributed to FBLIM1. A recent study by Cox et al. provides support for the notion that FBLIM1, a gene responsible for encoding a protein involved in bone remodeling, plays a crucial role in the development of sterile bone inflammation. Through whole-exome sequencing, researchers successfully identified a homozygous mutation within the filamin-binding region of FBLIM1 in an affected child who had parents with a blood relation [15]. Unfortunately, there is limited information on the role of FBLIM1 in cancers. According to the findings of a recent study, FBLIM1 is abundantly expressed in oral cancer [16]. Furthermore, inhibiting FBLIM1 was revealed to hinder oral cancer cell proliferative, migratory, and invasive properties via modulating the pathway that is controlled by EGFR. It has not been determined, to the best of our knowledge, whether FBLIM1 was expressed or functions in gliomas.

Many different cell components make up a solid tumor. These cell components include fibroblasts, macrophages, lymphocytes stromal cells, endothelial cells, cancer cells, hematopoietic cells, and smooth muscle cells [17,18]. The most indispensable components required for the creation and development of a tumor are immune cells and stromal cells, among other cell types [19]. How the components of tumor microenvironment (TEM) interact with one another and behave is of the utmost importance to the analysis of tumors [20,21]. Recent research has demonstrated that both stromal cell activity and its interaction with tumor cells contribute to tumor growth, invasion of surrounding tissue, and dissemination throughout the body [22,23]. In addition, the stromal cells have the potential to secrete cytokines, chemokines, and growth factors, all of which notably impact the features of the tumor. Because of the strong connection between the growth of LGG and immunity, LGG cells have the ability to generate a significant number of cytokines. These cytokines encourage diverse immune cells to enter the tumor, which accordingly provides a TME. Glioma patients who have poor prognosis are closely related to immune cells [24,25]. Tumor-associated macrophages (TEM) are implicated in glioma development, relapse, aggressiveness, and the response to therapy. Glioma patients often face a poor prognosis. Given the unique advantages it offers in the treatment of various malignancies, immuno-oncology is currently attracting significant clinical attention. Immune-infiltrating cells and immune-related genes both play crucial roles within the TEM, contributing to the determination of patient prognosis and providing strong rationale for immunotherapy.

In this study, we aimed to explore the expression, clinical significance, and potential function of FBLIM1 in glioma patients. First, we compared the differential FBLIM1 expression in normal tissues and glioma samples using the glioma RNA-seq data provided by TCGA and CGGA. Then, we explored the connection between the levels of FBLIM1 expression and the clinical pathological characteristics of glioma. In addition, we investigated the potential role that FBLIM1 plays in glioma prognosis. In addition, we carried out gene enrichment analysis to investigate and reveal its potential functions. In the end, we deciphered the connection between immune infiltration and FBLIM1 expression, as well as its mechanism in the process of developing and promoting glioma. In summary, this study provided valuable insights into the biological characteristics of gliomas and potential therapeutic targets. By elucidating the expression, clinical significance, potential functions, and its association with immune infiltration of FBLIM1, this research had the potential to offer new insights and strategies for the diagnosis and treatment of gliomas. These findings may have a positive impact on improving patient prognosis and survival rates.

## 2 Materials and methods

### 2.1 Cell culture and transfection

Glioma cell lines (normal human astrocyte (NHA), U251, and LN229 cells) and NHA cells were acquired from the American Type Culture Collection (Manassas, VA, USA) and cultivated with 10% fetal bovine serum-contained Dulbecco’s modified Eagle’s medium. SiRNA targeting FBLIM1 as well as controls devoid of target sequence were procured from Shanghai GenePharma Co., Ltd. Using the protocol provided by the manufacturer of Lipofectamine^®^ 2000 (Invitrogen; Thermo Fisher Scientific, Inc., CA, USA), siRNAs were introduced into cells via transfection using this product.

### 2.2 RNA isolation, reverse transcription, and qPCR

The isolation of the total RNA was achieved by following the protocol provided by the manufacturer of the TRIzol reagent (Invitrogen, Carlsbad, CA, USA) and using their product. PrimeScript RT Master Mix (Takara, Japan) was implemented to carry out the process of reverse transcription of mRNAs. qPCR was carried out with the use of SYBR Green (Takara, Japan) in accordance with the guidelines provided by the manufacturer. The findings were standardized with respect to the expression of GADPH, and the calculations were carried out using the 2-Cq technique. All experiments were in triplicate. Primer sequences were as follows: FBLIM1 forward: 5′-TGTAGCCGTGAGTGAGGAAGT-3, FBLIM1 reverse: 5′-CAGGTGTCTTTGTGGGAAGCA-3′; GAPDH forward: 5′-GGAGCGAGATCCCTCCAAAAT-3′, GAPDH reverse: 5′-GGCTGTTGTCATACTTCTCATGG-3′.

### 2.3 Cell proliferation assays

Cell proliferation was determined using a Cell Counting Kit-8 (CCK-8, Beyotime Institute of Biotechnology, Shanghai, China) according to the manufacturer’s instructions. For the CCK-8 assay, 2 × 10^4^ cells/well were seeded in a 96-well plate for 24 h and were transiently transfected with si-FBLIM1 or scramble siRNA. Subsequently, at multiple time points (0, 1, 2, and 3 days after transfection), 10 microliters of the Cell Counting Kit solution were added to each well. This solution contains a tetrazolium salt (WST-8) that is reduced by cellular dehydrogenases to formazan in viable cells. The 96-well plate was then incubated for 2 h at 37°C to allow the CCK-8 reagent to interact with the cells. Following incubation, the absorbance values at each time point were measured at 450 nm using a microplate reader. The absorbance values obtained were directly proportional to the number of viable cells in each well and were used to assess cell proliferation over time. To ensure the reliability of our findings, all experiments were biologically repeated a minimum of three times.

### 2.4 Data sets

The RNA-seq data of LGG samples, at level 3, as well as clinical data, were acquired from the UCSC Xena1 database. By using a genome tissue expression (GTEx), researchers were able to collect data on gene expression in normal tissues. Normalized gene expression was assessed using the Log2-based transformation, with the fragments per kilobase of transcript per million mapped reads as units of measurement. Then, the “sva” package of the R program was employed to normalize the RNA expression profiles and eliminate the batch effects. Principal component analysis was used for both the GTEx and TCGA datasets (https://portal.gdc.cancer.gov/) in order to identify batch effects. In addition, we accessed the CGGA database (http://cgga.org.cn/) and retrieved the RNA-Seq and clinical information of 749 glioma samples. In the TCGA database, we selected a dataset relevant to gliomas. We verified that the chosen dataset includes clinical information such as patient age, gender, survival time, and RNA-seq data from glioma tissues. Depending on the research questions, further sample selection was conducted, such as filtering for specific subtypes of gliomas or samples related to specific treatment regimens. For the CGGA database, we also selected a dataset related to gliomas and ensured that it included both clinical information and transcriptome data from glioma tissues. For clinical data, we conducted data cleaning and standardization, handling missing values and outliers. Additionally, we created relevant clinical features based on the characteristics of gliomas, such as staging and tumor size. For transcriptome data, quality control procedures were implemented to exclude samples of poor quality. We applied normalization methods to ensure data comparability and performed gene expression filtering or transformations as needed.

### 2.5 FBLIM1 differential expression in glioma tissues

In order to calculate the differential expression of FBLIM1, boxplots, and scatter plots were produced with the disease state as the variable. The disease state in question was either tumor or normal. Receiver operating characteristic (ROC) curves were employed in order to estimate the diagnostic performance of FBLIM1. The statistical ranking for FBLIM1 expression that was high or low, respectively, was characterized as FBLIM1-high or FBLIM1-low depending on whether it was above or below the median value.

### 2.6 Association of FBLIM1 with prognosis

The overall survival (OS), disease-specific survival (DSS), and progression-free interval (PFI) were key assessment factors for the relevance between FBLIM1 expression and the glioma’s prognosis. mRNA expression levels were employed in conjunction with the log-rank test to determine survival rates, which were estimated by the Kaplan–Meier plot. The parameters that were chosen were as follows: mRNA (RNA-seq) for pan-cancer, and patients were divided by their median age. To address the importance of FBLIM1 mRNA expression in glioma from a predictive standpoint, we performed univariate and multivariate Cox analyses based on the TCGA dataset. The cut-off value was determined to be the level of FBLIM1 mRNA expression that was found in the median. The independent predictive significance of FBLIM1 mRNA expression levels was subsequently validated by using multivariate Cox analysis. Multivariate Cox analysis is a statistical technique used in survival analysis and epidemiology. It extends the traditional univariate Cox proportional hazards regression by allowing the simultaneous analysis of multiple independent variables (covariates) to assess their impact on the time to an event, typically a survival time or time to a specific outcome, while accounting for censoring.

### 2.7 Identification of differently expressed genes (DEGs) between FBLIM1-low and -high expression glioma groups

DEGs between samples with low FBLIM1 and high FBLIM1 expression from the TCGA database were evaluated with DESeq2 (4.0) software using the Student’s *t*-test. It was determined that genes met the criterion for statistical significance when the absolute log (FC) was >2, and the adj *p*-value <0.05.

### 2.8 Functional enrichment analysis

ClusterProfiler is an R package used for bioinformatics and biological data analysis. It is employed for functional enrichment analysis and visualization of high-throughput biological data. ClusterProfiler assists researchers in understanding the biological significance of gene sets, particularly in high-throughput gene expression analysis [26]. For functional enrichment analysis, the ClusterProfiler package of the R program was utilized, and the GO biological processes and KEGG pathways that met the criteria for significance (*q*-value of at least 0.01) were utilized. In addition, the threshold conditions were a significance level of *p*-value < 0.05 and an adjusted *p*-value < 0.05.

### 2.9 Immune infiltration analysis

The infiltration of 24 distinct types of immune cells into glioma samples was examined with the Spearman correlation analysis. These immune cell types were identified following the published immunocytes characteristics. SsGSEA (single-sample Gene Set Enrichment Analysis) is a method used in bioinformatics and biological data analysis to assess the enrichment of gene sets in individual samples. It is developed based on the traditional gene set enrichment analysis (GSEA) and is commonly used for analyzing high-throughput gene expression data from single samples, such as RNA-seq data. This analysis was implemented by ssGSEA in glioma samples with the GSVA package. Scatter plots illustrate the relationship that exists between the expression of FBLIM1 and certain immune cells. The enrichment score of each immunocyte was compared between the FBLIM1-high samples and the FBLIM1-low samples using the Wilcoxon rank-sum test.

### 2.10 Statistical methods

R software (4.0.2, Boston, Massachusetts, USA) or GraphPad Prism 6 (GraphPad Prism, San Diego, CA, USA) were used for all analyses. Statistical differences between groups were evaluated using the Student’s paired two-tailed *t*-test. Adjusted *p*-values < 0.05, *p*-values < 0.05, and FC > 2 were utilized as statistical thresholds.

## 3 Results

### 3.1 FBLIM1 mRNA expression was distinctly upregulated in glioma

**Figure 1:**
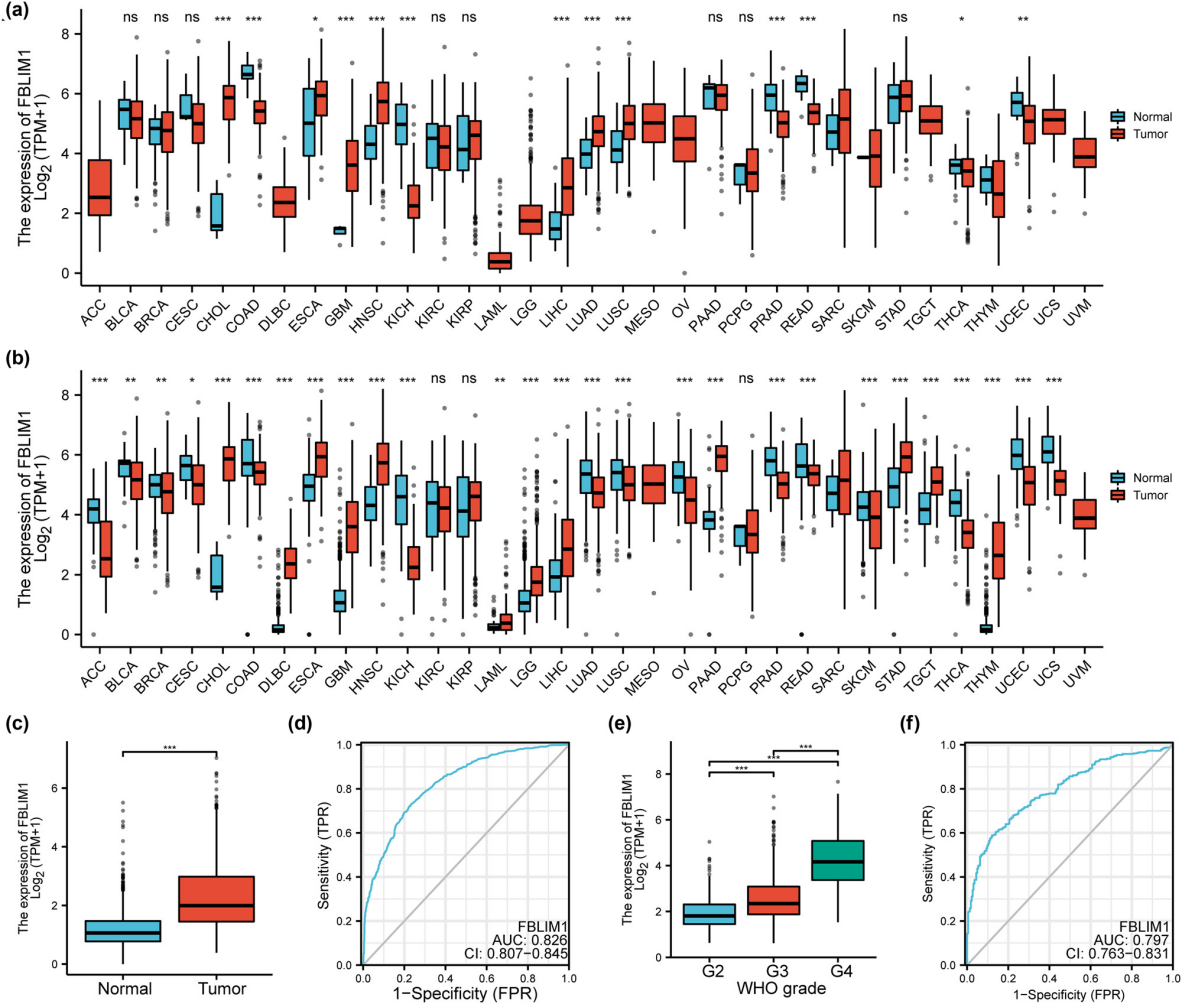
FBLIM1 expression was distinctly increased in glioma. (a and b) FBLIM1 expression levels in various cancer types from TCGA data and GTEx. (c) FBLIM1 levels in glioma and normal brain specimens. (d) A ROC curve was created for testing the importance of FBLIM1 for identifying glioma tissues. (e) FBLIM1 expression in glioma specimens with different clinical stages. (f) ROC assays were employed to examine the diagnostic power of FBLIM1 levels in screening glioma specimens with G3–G4 from glioma specimens with G2.

Initially, FBLIM1 mRNA expression in the tumor samples of GTEx coupled with TCGA and the corresponding normal samples of TCGA were compared for the pan-cancer analysis by the Wilcoxon rank sum test. As shown in Figure 1a and b, we observed that FBLIM1 exhibited a dysregulated level in many types of tumors. However, the expression trend of FBLIM1 was different in different tumors, suggesting that it may serve as a tumor promotor or a tumor suppressor according to the types of tumors. Importantly, we observed that FBLIM1 expression was notably elevated in glioma specimens versus non-tumor specimens (Figure 1c). To further explore the diagnostic value of FBLIM1 expression for glioma, we performed ROC assays and found that FBLIM1 could effectively distinguish glioma specimens from normal tissue samples (AUC: 0.826; 95% CI: 0.807–0.845; *p* < 0.001, Figure 1d). Moreover, FBLIM1 expression was revealed to be distinctly increased in GBM specimens compared with LGG specimens (Figure 1e). Meanwhile, the findings of ROC assays also substantiated the diagnostic value of FBLIM1 in screening GBM specimens from LGG specimens with an AUC of 0.797 (Figure 1f).

### 3.2 Association with FBLIM1 expression and clinicopathological factors

**Figure 2:**
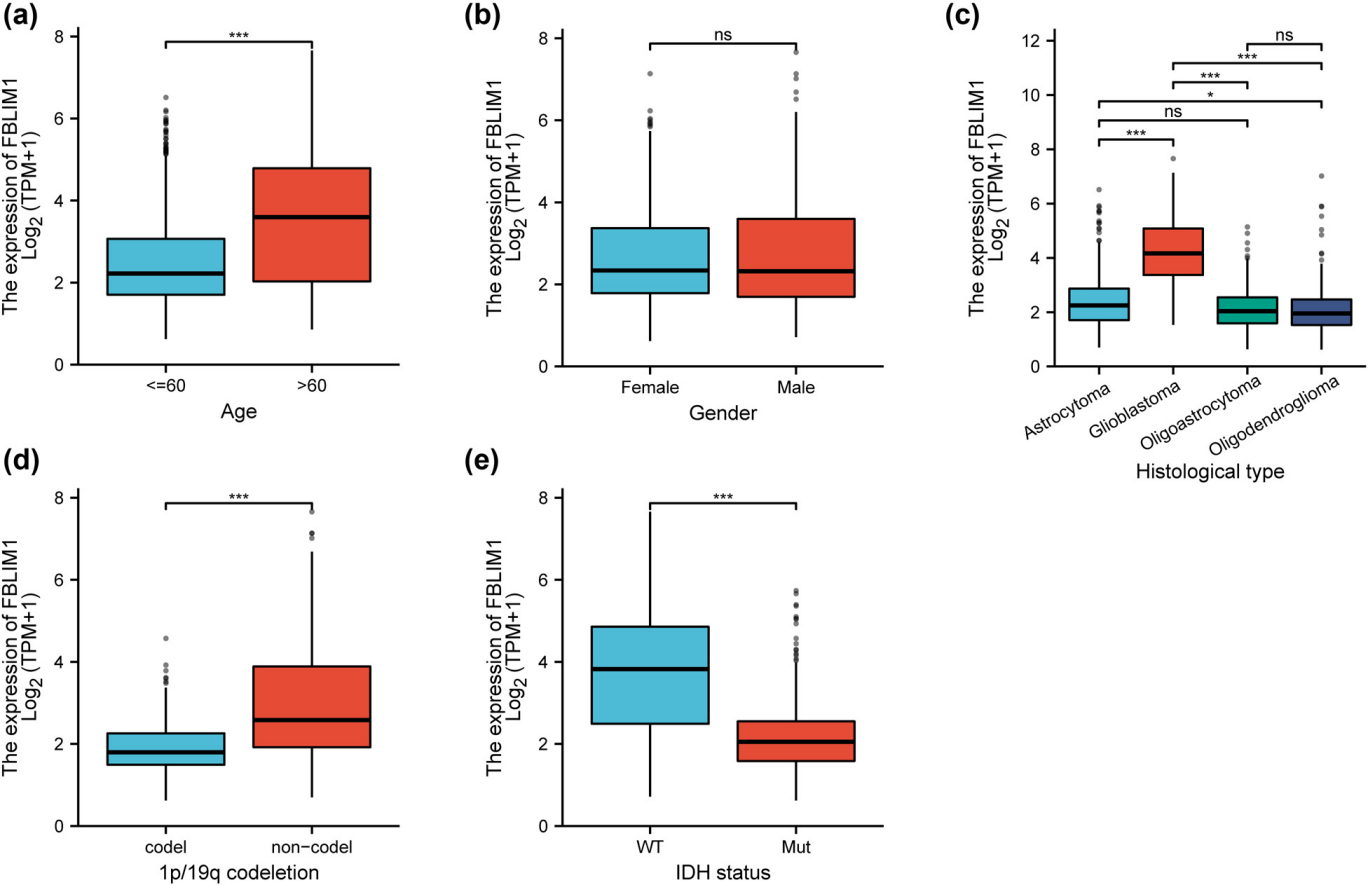
Relation with FBLIM1 levels and clinicopathological traits, consisting of (a) gender, (b) age, (c) histological type, (d) 1p/19q codeletion, and (e) IDH status.

We estimated the possible link between FBLIM1 expression and clinical factors. FBLIM1 expression did not show a distinct difference between female patients and male patients (Figure 2a), while it showed a higher level in patients with age >60 in contrast to those with age <60 (Figure 2b). Moreover, GBM patients showed a higher level of FBLIM1 (Figure 2c). In addition, there was a dysregulated level of FBLIM1 in glioma patients with different 1p/19q codeletion (Figure 2d) and IDH status (Figure 2e). For statistical analysis, the patients were classified into the FBLIM1 high-expression (*n* = 348) and low-expression groups (*n* = 348) based on the mean value of FBLIM1 expression. In order to explore the association between FBLIM1 expression and clinicopathologic features in glioma, we manually divided glioma patients into two groups (high and low-expression groups) based on the mean expression of FBLIM1. Table 1 shows that FBLIM1 expression had a relation with age, histological type, WHO grade, 1p/19q codeletion, IDH status, and primary therapy outcome (all *p* < 0.05). Our findings suggested the essentiality of FBLIM1 in the clinical progression of glioma patients.

**Table 1:** Correlation between FBLIM1 and clinicopathological parameters

Characteristics	Low expression of FBLIM1	High expression of FBLIM1	*p*
*n*	348	348	
Age, *n* (%)			<0.001
≤60	303 (43.5%)	250 (35.9%)	
>60	45 (6.5%)	98 (14.1%)	
Gender, *n* (%)			0.939
Female	148 (21.3%)	150 (21.6%)	
Male	200 (28.7%)	198 (28.4%)	
Histological type, *n* (%)			<0.001
Astrocytoma	109 (15.7%)	86 (12.4%)	
Glioblastoma	10 (1.4%)	158 (22.7%)	
Oligoastrocytoma	88 (12.6%)	46 (6.6%)	
Oligodendroglioma	141 (20.3%)	58 (8.3%)	
WHO grade, *n* (%)			<0.001
G2	171 (26.9%)	53 (8.3%)	
G3	121 (19.1%)	122 (19.2%)	
G4	10 (1.6%)	158 (24.9%)	
IDH status, *n* (%)			<0.001
WT	58 (8.5%)	188 (27.4%)	
Mut	288 (42%)	152 (22.2%)	
1p/19q codeletion, *n* (%)			<0.001
Codel	134 (19.4%)	37 (5.4%)	
Non-codel	214 (31.1%)	304 (44.1%)	
Primary therapy outcome, *n* (%)			<0.001
PD	49 (10.6%)	63 (13.6%)	
SD	98 (21.2%)	49 (10.6%)	
PR	41 (8.9%)	23 (5%)	
CR	102 (22.1%)	37 (8%)	
Age, median (IQR)	41 (33, 52.25)	52 (37, 62)	<0.001

Bold values indicates the significance values (*p*).

### 3.3 FBLIM1 expression is survival-associated

**Figure 3:**
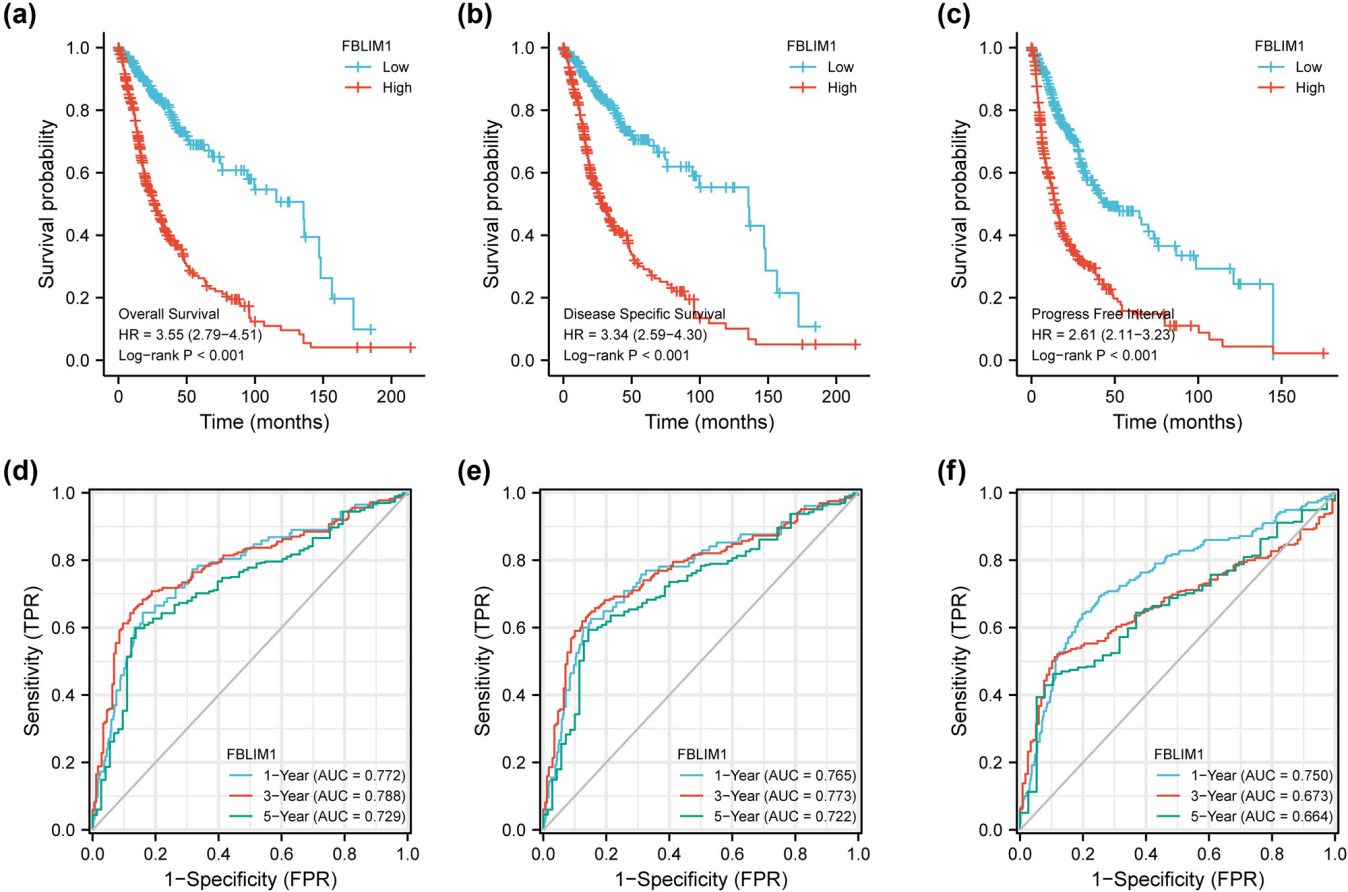
Influences of differential FBLIM1 levels on the glioma patients’ prognosis. (a–c) Kaplan–Meier curves of OS, DSS, and PFI between the FBLIM1-high and -low level cohorts. (d–f) ROC curves of 1-, 3-, and 5-year OS, DSS, and PFI.

**Figure 4:**
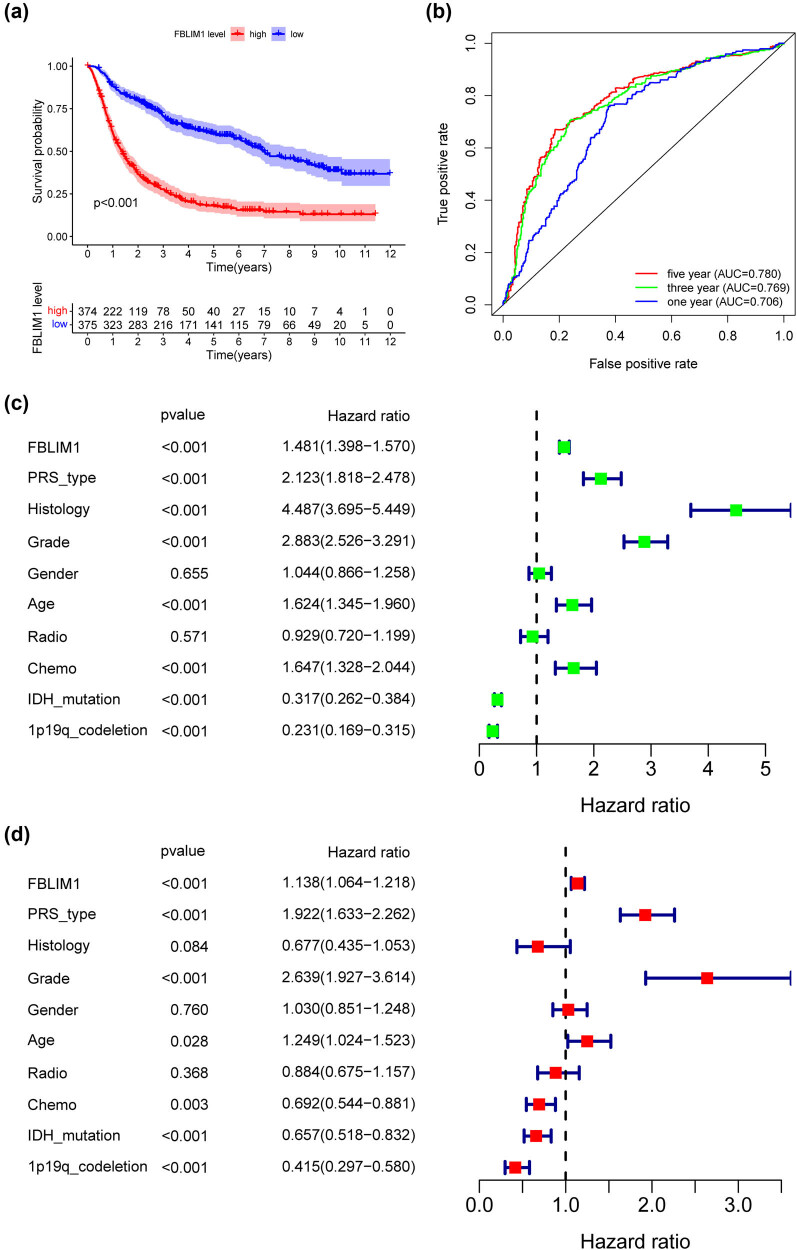
The prognostic value of FBLIM1 levels in glioma patients using CGGA datasets (*n* = 749). (a) Kaplan–Meier curves for OS in glioma patients with glioma grouped following FBLIM1 expression. (b) ROC curve analysis of FBLIM1. (c and d) Univariate and multivariate analysis of FBLIM1.

**Figure 5:**
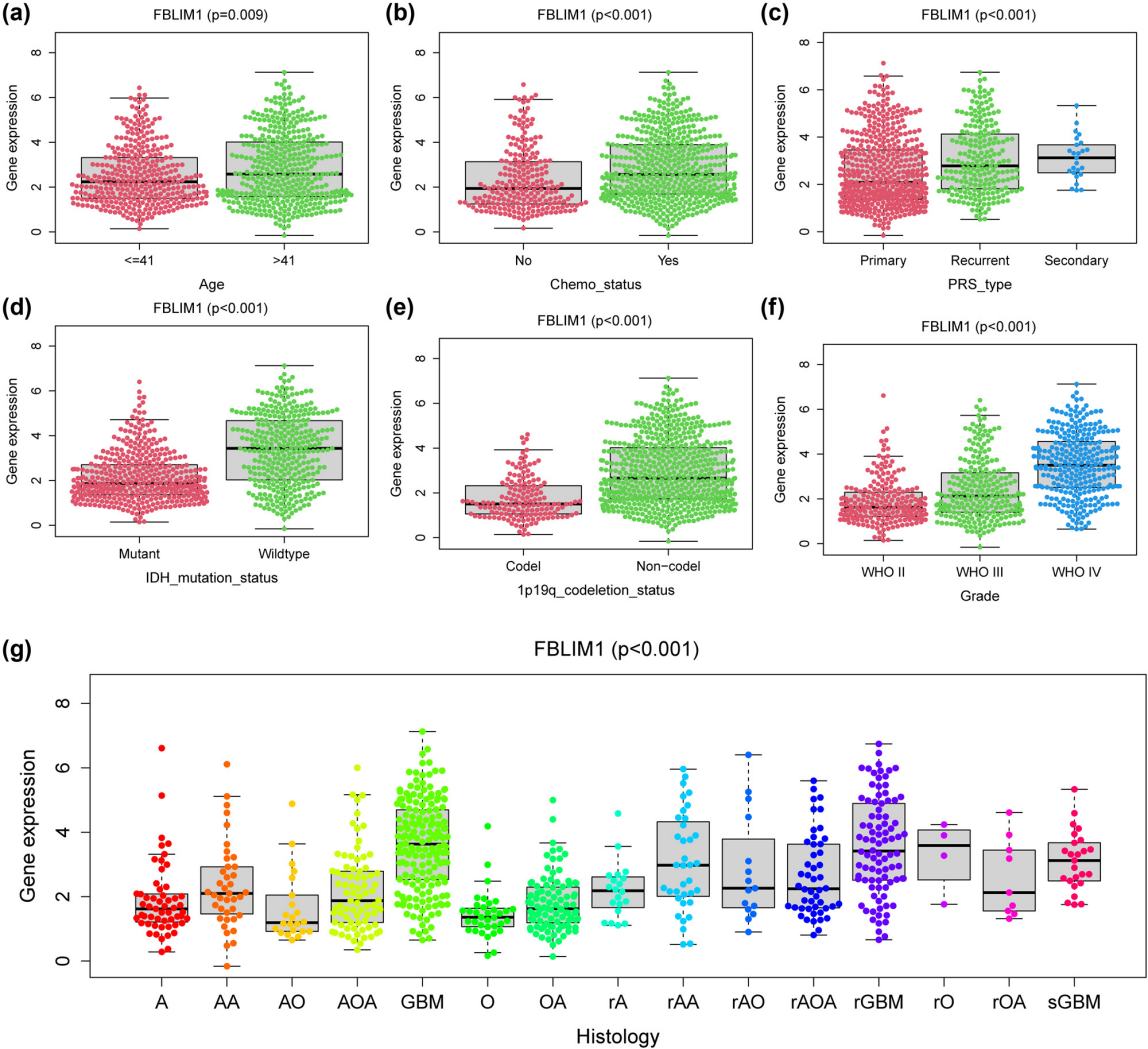
Relevance with FBLIM1 levels and clinicopathological traits, consisting of (a) age, (b) Chemo_status, (c) PRS_type, (d) 1p19q_codeletion_status, (e) IDH_mutation_status, (f) grade, and (g) histology.

The cohorts contained the low-and high-expression subgroups following the median expression of FBLIM1. On the basis of the KM plot in TCGA, patients with high FBLIM1 expression exhibited shorter OS (Figure 3a), DSS (Figure 3b), and PFI (Figure 3c) in comparison to those with low FBLIM1 expression. Moreover, the predictive power of FBLIM1 expression was estimated by the ROC analysis, which unveiled that FBLIM1 expression could, respectively, forecast the 1-year (0.772), 3-year (0.788), and 5-year (0.729) survival of glioma patients (Figure 3d) in an effective way. In addition, a similar result was witnessed in DSS (Figure 3e) and PFI (Figure 3f). We further performed multivariate assays to validate the prognostic value of FBLIM1 expression and observed that FBLIM1 expression was a prognostic factor for OS (*p* = 0.032, Table 2) and PFI (*p* = 0.005, Table 3) while DSS (*p* = 0.151, Table 4) was not. On the other hand, the prognostic power of the FBLIM1 level was assessed in glioma patients from the CGGA datasets. Patients with high FBLIM1 levels exhibited a shorter OS in contrast to those with a low level (Figure 4a). Time–ROC assays also confirmed the strong ability to forecast the clinical outcomes of glioma patients (Figure 4b). Importantly, multivariate assays also validated that FBLIM1 level was regarded as a prognostic factor for OS for glioma patients (Figures 4c and d). We also observed based on the CGGA datasets that FBLIM1 level was linked to age, IDH_mutation_status, Chemo_status, PRS_type, 1p19q_codeletion_status, grade, and histology (Figure 5a–g).

**Table 2:** Univariate and multivariate Cox’s Hazard analysis on possible prognostic factors for OS of glioma patients

Characteristics	Total (*N*)	Univariate analysis		Multivariate analysis	
		Hazard ratio (95% CI)	*p* value	Hazard ratio (95% CI)	*p* value
Age	695				
≤60	552	Reference			
>60	143	4.668 (3.598–6.056)	**<0.001**	1.893 (1.409–2.545)	**<0.001**
Gender	695				
Female	297	Reference			
Male	398	1.262 (0.988–1.610)	0.062	1.331 (1.013–1.747)	**0.040**
WHO grade	634				
G2	223	Reference			
G3 and G4	411	5.642 (3.926–8.109)	**<0.001**	2.069 (1.363–3.139)	**<0.001**
IDH status	685				
WT	246	Reference			
Mut	439	0.117 (0.090–0.152)	**<0.001**	0.221 (0.153–0.320)	**<0.001**
1p/19q codeletion	688				
Codel	170	Reference			
Non-codel	518	4.428 (2.885–6.799)	**<0.001**	1.418 (0.851–2.362)	0.180
FBLIM1	695				
Low	347	Reference			
High	348	3.581 (2.724–4.709)	**<0.001**	1.444 (1.033–2.020)	**0.032**

Bold values indicates the significance values (*p*).

**Table 3:** Univariate and multivariate Cox’s Hazard analysis on possible prognostic factors for progress free interval of glioma patients

Characteristics	Total (*N*)	Univariate analysis		Multivariate analysis	
		Hazard ratio (95% CI)	*P* value	Hazard ratio (95% CI)	*P* value
Age	695				
<=60	552	Reference			
>60	143	2.873 (2.268–3.640)	**<0.001**	1.255 (0.957–1.644)	0.100
Gender	695				
Female	297	Reference			
Male	398	1.083 (0.875–1.342)	0.463		
WHO grade	634				
G2	223	Reference			
G3&G4	411	2.751 (2.112–3.583)	**<0.001**	1.191 (0.870–1.632)	0.276
IDH status	685				
WT	246	Reference			
Mut	439	0.151 (0.119–0.191)	**<0.001**	0.206 (0.149–0.283)	**<0.001**
1p/19q codeletion	688				
Codel	170	Reference			
Non-codel	518	3.373 (2.438–4.666)	**<0.001**	1.332 (0.906–1.959)	0.145
FBLIM1	695				
Low	347	Reference			
High	348	2.640 (2.112–3.301)	**<0.001**	1.482 (1.127–1.949)	**0.005**

Bold values indicates the significance values (*p*).

**Table 4:** Univariate and multivariate Cox’s hazard analysis on possible prognostic factors for disease-specific survival of glioma patients

Characteristics	Total (*N*)	Univariate analysis		Multivariate analysis	
		Hazard ratio (95% CI)	*p* value	Hazard ratio (95% CI)	*p* value
Age	674				
≤60	541	Reference			
>60	133	4.500 (3.409–5.940)	**<0.001**	1.792 (1.307–2.457)	**<0.001**
Gender	674				
Female	289	Reference			
Male	385	1.248 (0.965–1.614)	0.092	1.287 (0.961–1.723)	0.091
WHO grade	614				
G2	220	Reference			
G3&G4	394	5.816 (3.956–8.551)	**<0.001**	2.104 (1.349–3.283)	**0.001**
IDH status	664				
WT	232	Reference			
Mut	432	0.110 (0.083–0.146)	**<0.001**	0.203 (0.138–0.300)	**<0.001**
1p/19q codeletion	668				
Codel	169	Reference			
Non-codel	499	4.987 (3.117–7.978)	**<0.001**	1.571 (0.900–2.742)	0.112
FBLIM1	674				
Low	344	Reference			
High	330	3.367 (2.537–4.468)	**<0.001**	1.289 (0.911–1.824)	0.151

Bold values indicates the significance values (*p*).

### 3.4 Functional enrichment analysis of DEGs between FBLIM1-low and -high expression glioma groups

**Figure 6:**
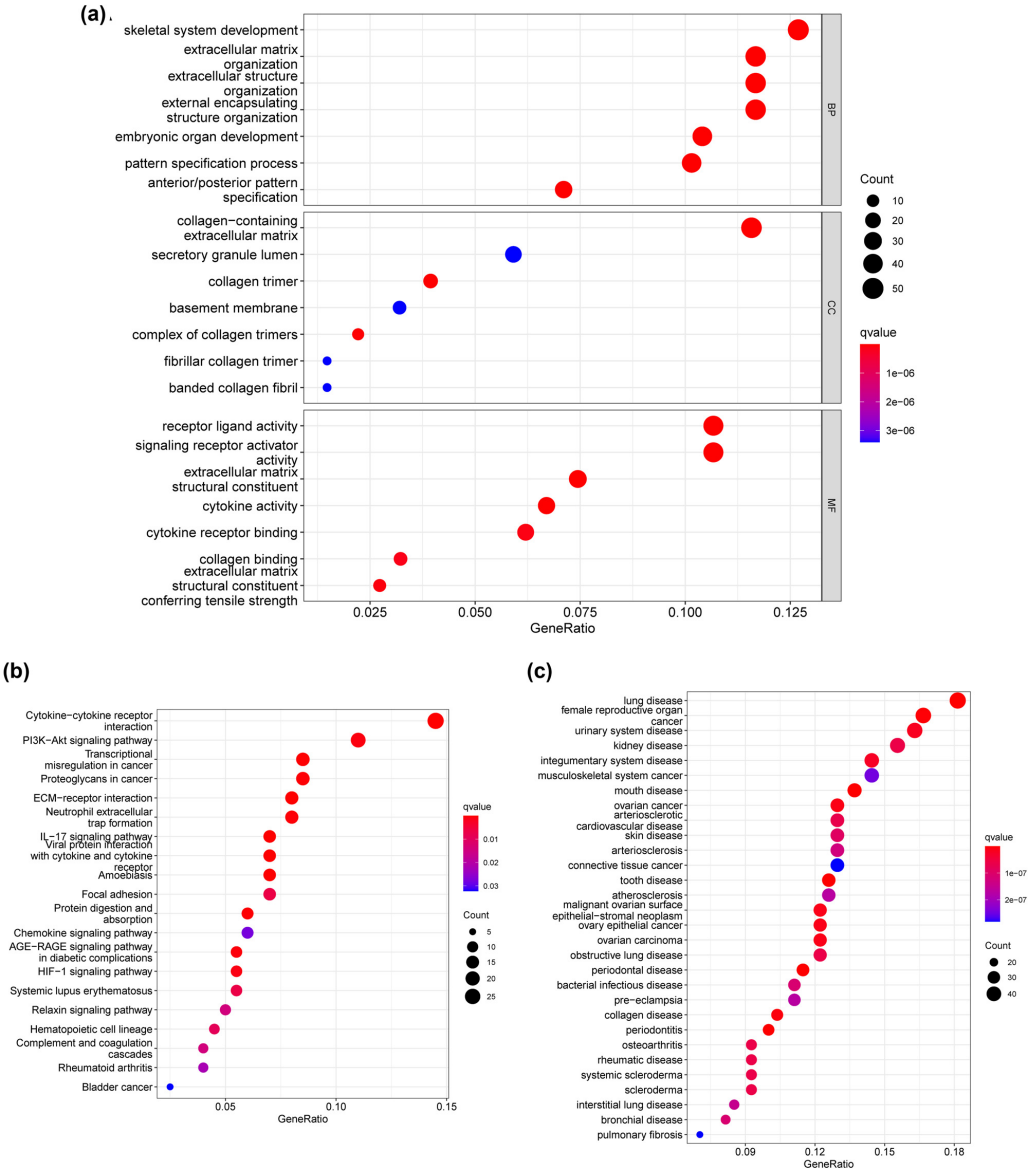
Functional enrichment analysis results. (a) GO analysis outcomes of DEGs, and the topmost seven terms of every category were displayed. (b) The topmost 20 pathways of KEGG analysis. (c) Disease ontology enrichment analysis.

To explore the possible function of FBLIM1 in glioma, we screened DEGs between FBLIM1-low and -high expression glioma groups, and 420 DEGs were identified. Then, we performed GO analysis and found that 420 DEGs were mainly associated with skeletal system development, secretory granule lumen, extracellular structure organization, extracellular matrix organization, extracellular matrix structural constituent, collagen trimer, receptor-ligand activity, collagen-containing extracellular matrix, and signaling receptor activator activity (Figure 6a). The results of KEGG indicated that 420 DEGs were majorly enriched in the PI3K-Akt signaling pathway, neutrophil extracellular trap formation, ECM–receptor interaction, proteoglycans in cancer, transcriptional misregulation in cancer, and cytokine–cytokine receptor interaction (Figure 6b). DO assays showed that 420 DEGs were mainly associated with lung disease, female reproductive organ cancer, urinary system disease, kidney disease, integumentary system disease, and musculoskeletal system cancer (Figure 6c).

### 3.5 FBLIM1 expression correlates to immune infiltration

**Figure 7:**
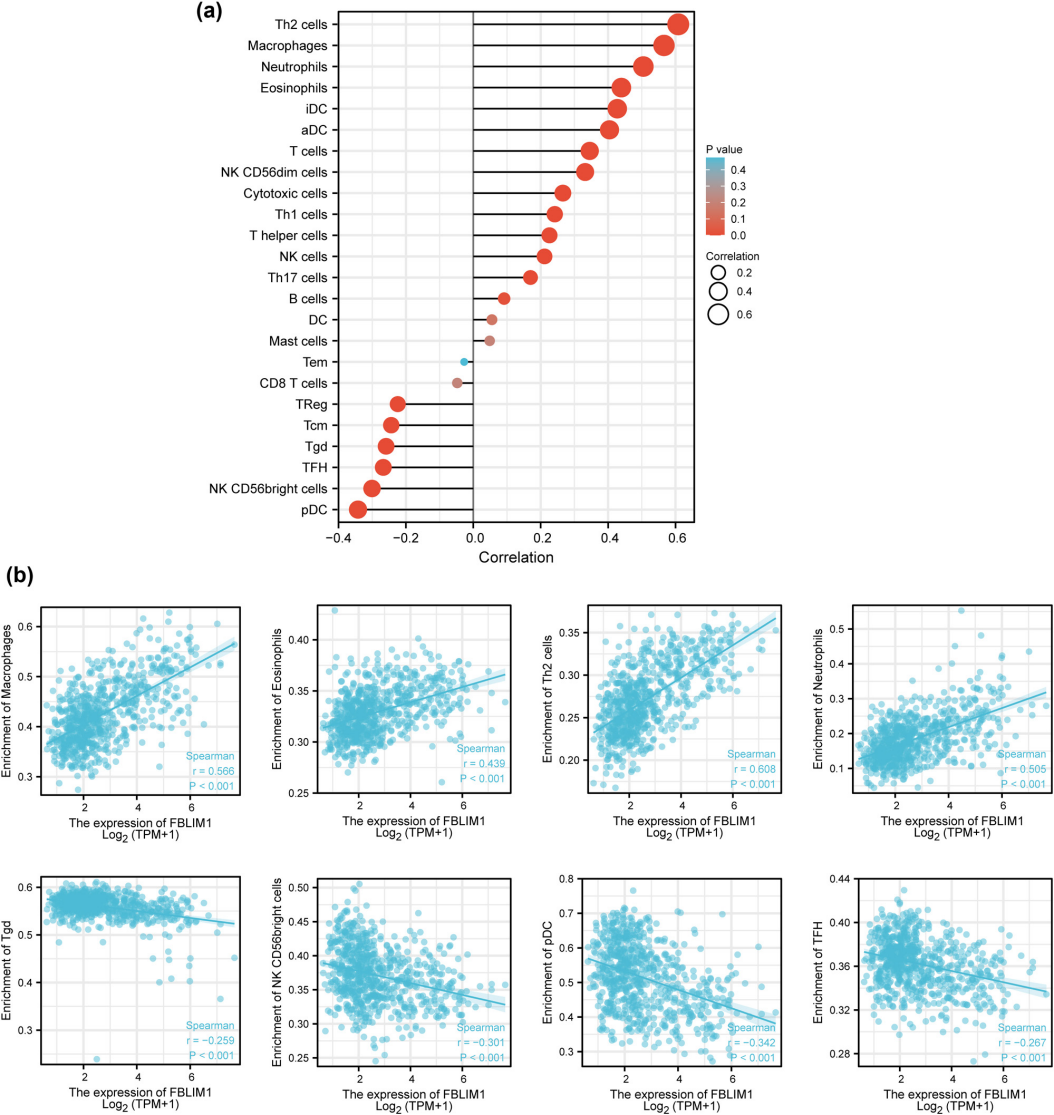
(a and b) FBLIM1 levels were linked to the immune infiltration in the TME.

The relevance between FBLIM1 expression and the ssGSEA-quantified level of immune cell infiltration was processed using Spearman’s correlation. FBLIM1 level was linked to many immune cells, including Th2 cells, macrophages, neutrophils, TFH, pDC, and NK CD56 bright cells (Figure 7a and b)

### 3.6 Oncogenic roles of FBLIM1 in glioma

**Figure 8:**
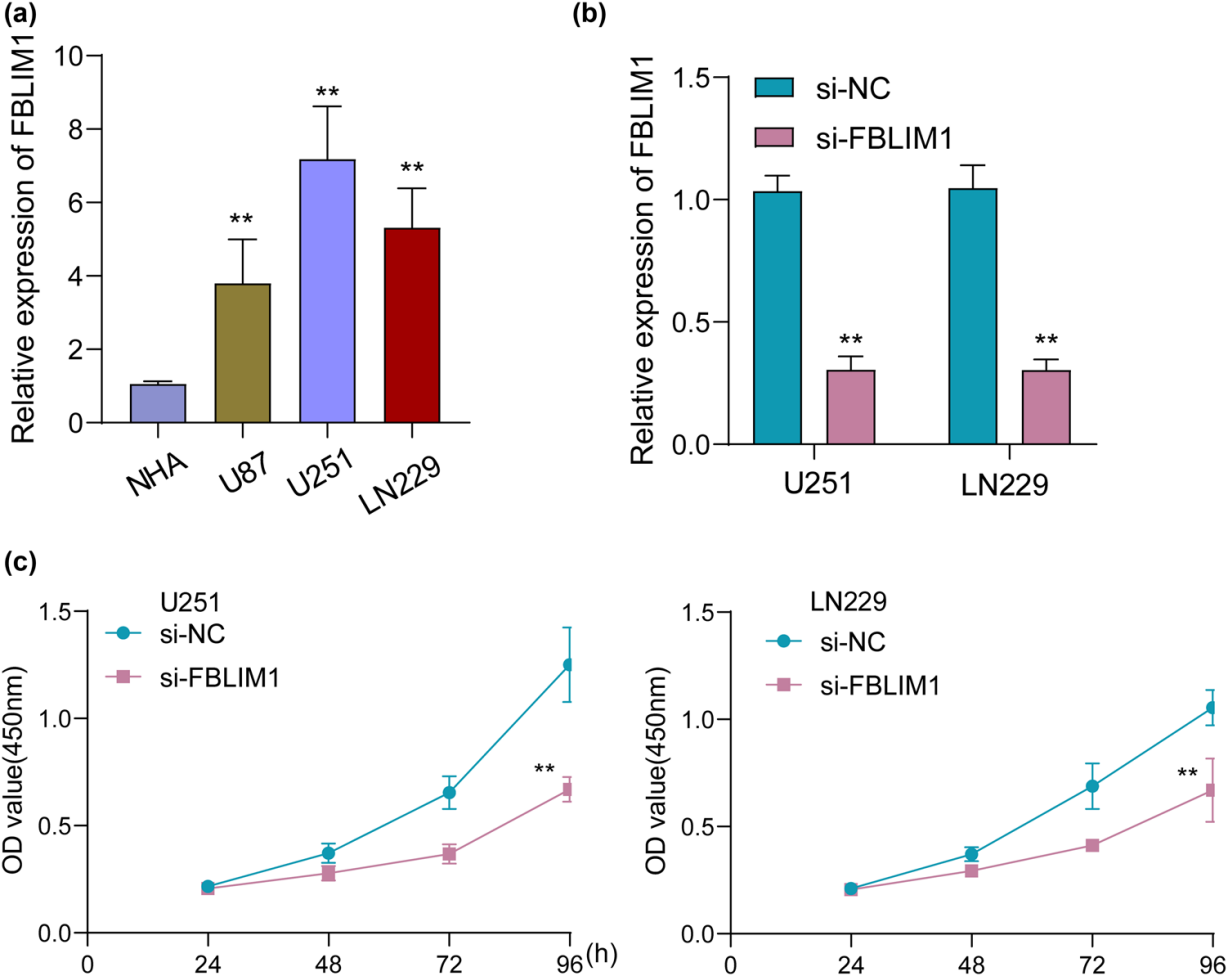
FBLIM1 knockdown suppressed glioma cell proliferation. (a) RT-PCR was applied to examine FBLIM1 levels in three glioma cells and NHA cells. (b) FBLIM1 expression was decreased in glioma after the transfection of si-FBLIM1. (c) CCK-8 analysis was employed for elucidating the function of FBLIM1 knockdown on U251 and LN229 cell proliferation.

To examine FBLIM1 expression in glioma, we performed RT-PCR and found that the FBLIM1 level was distinctly elevated in three glioma cells compared with normal brain cells (Figure 8a). In addition, the si-FBLIM1’s transfection efficiency in U251 and LN229 cells was further demonstrated by RT-PCR (Figure 8b). Finally, we performed CCK-8 assays and observed that the knockdown of FBLIM1 distinctly suppressed the proliferation of U251 and LN229 cells (Figure 8c).

## 4 Discussion

High mortality and disability rates are associated with glioma, which is the most frequent primary brain tumor seen in adults and has a high incidence rate [27]. At present, surgical intervention, postoperative adjuvant chemotherapy, and radiation therapy are the cardinal treatment for glioma [28]. Even when using a variety of different therapy approaches at the same time, the prognosis for glioma patients is currently quite dismal [29]. The identification of prognostic molecular biomarkers that might give explanations about glioma development and progression has been the subject of a great number of significant attempts in recent years [30,31]. Targeted therapy, aiming to specifically target genes within tumor cells to improve patient prognoses, is promising for innovative approaches to eradicate glioma cells and expand treatment possibilities. Consequently, the exploration of novel molecular targets that prove effective in the diagnosis and treatment of glioma is of utmost importance.

Recently, several studies have focused on molecular targeted therapy, leading to the identification of genes associated with the diagnosis, treatment, and prognosis of glioma. For instance, Jiang et al. reported that the restoration of CLCF1 is associated with immunosuppression and poor prognosis in gliomas, highlighting its significant prognostic value. CLCF1 may potentially serve two important roles as a promising target linked to immunotherapy outcomes: suppressing tumor development and synergizing with immunotherapy [32]. Sun et al. found that in comparison to the controls, high-grade gliomas, and IDH wild-type gliomas exhibited a distinct elevated MXRA5 level. The ROC findings found that MXRA5 has the potential to function as an indicator for the mesenchymal subtype of glioblastoma multiforme. The expression of MXRA5 was shown to have a strong correlation with the levels of expression of immune checkpoint molecules and macrophage infiltration associated with tumors. High MXRA5 expression might act as an independent signal of a bad prognosis in glioma [33]. However, many glioma-related genes have not been investigated. In our work, we found a novel glioma-related gene FBLIM1, which is highly expressed in many types of tumors, including glioma. Previously, a study reported that when compared with their normal counterparts, OSCC-derived cell lines and actual OSCC specimens exhibited much higher levels of FBLIM1 expression than their normal counterparts did. Also, a correlation was found between the expression of FBLIM1 and the size of the main tumor and its vascular invasion. Oral cancer cells were much less able to proliferate, migrate, and invade when FBLIM1 was knocked down, and this suppression was achieved by modification of the EGFR pathway. Based on these findings, FBLIM1 may be an oncogene that contributes to the development of oral cancer. We highlighted that high FBLIM1 expression had relevance with poor clinical prognosis in both TCGA and CGGA datasets. FBLIM1 might be a prognostic index in glioma following the univariate and multivariate Cox analyses. Based on functional enrichment analysis, FBLIM1 may play multiple significant roles in glioma. These DEGs covered a wide range of functional areas, including skeletal system development, extracellular matrix structure, and cell signaling pathways. KEGG analysis indicated that these DEGs are enriched in several key signaling pathways, such as the PI3K-Akt signaling pathway, immune-related pathways, and cancer-related pathways, suggesting their potential involvement in glioma growth, infiltration, and immune responses. Furthermore, DO analysis revealed that these DEGs are associated with various diseases and disease systems, including cancer, urinary system diseases, and lung diseases. These findings suggest that these FBLIM1 may not only have critical functions in glioma but may also be relevant to other diseases. In addition, we also provided evidence that the knockdown of FBLIM1 distinctly suppressed the biological functions of glioma cells. Collectively, our study underlined that FBLIM1 was elevated in glioma, and FBLIM1 had prognostic power in glioma, signifying that glioma had vital modulatory functions in gliomas.

Notably, TME plays a part in tumor development. There are two types of solid tumors, namely hot tumors and cold tumors, which may be categorized immunologically [34,35]. Cancer immunotherapy is only effective in hot tumors but not cold tumors. Immunologically cool tumors are distinguished by a low mutation burden, a low immune cell infiltration, and larger numbers of myeloid-derived suppressor cells [36]. Based on this, immunologically cold tumors have worse clinical responses to the blocking of immune checkpoints. Yet, preliminary research has indicated that cool tumors may be converted into hot tumors. The development of a method to transform immunologically cool tumors into hot tumors depends on understanding the underlying mechanism of these conditions [37,38]. Tumor immunotherapies, including chimeric antigen receptor T-cell immunotherapy and anti-PD-1/PD-L1/CTLA-4 monoclonal antibodies, have received a lot of focus as an integral aspect of combination therapy in recent years [39]. Unlike targeted therapies or chemotherapy, immunotherapy has unique properties, which does not directly target cancer cells. In contrast, this treatment, through the antigen–antibody response, recruits and activates central immune-protective T cells to detect and kill cancer cells. Sadly, immunotherapy is not successful in treating every patient, particularly glioma patients. In this study, we found that the expression of FBLIM1 was positively associated with Th2 cells, macrophages, neutrophils, eosinophils, iDC, aDC, and T cells, while negatively associated with pDC, TFH, Tgd, Tcm, and NK CD56 bright cells. Tumor-associated macrophages, often featured with activated macrophages, are pivotal components of the TEM and linked to poor prognosis in other types of tumors. Surprisingly, our research revealed that a large macrophage and neutrophil infiltration was linked to a poor outcome. The intricacy of the TEM is shown by the fact that various cell types in the TEM can certainly impact the creation of invadopodium and intravasation by tumor cells. More research is required to fully understand the connection between FBLIM1 expression and immunoregulatory cells.

Several limitations are also demonstrated in our study. Initially, because the current research was carried out based on RNA sequences retrieved from a single database, and because the comprehensiveness of the data cannot be ensured, controlled and multi-center studies are necessary. Second, tumor tissues from the TCGA database had a larger number than normal tissues. Third, RNA sequencing data were the primary foundation for most of our findings. Due to the absence of information on the expression levels of other proteins except for FBLIM1, we were unable to investigate the direct mechanism by which FBLIM1 contributes to the progression of glioma.

## 5 Conclusion

Overall, FBLIM1 mRNA was shown to be significantly expressed in glioma, and its potential as a predictor of a positive result for glioma is being investigated. In glioma, it plays a part in the control of tumor promotor pathways as well as the infiltration of immune cells by tumoral tissue. The current findings point to the possibility that FBLIM1 mRNA might be employed as a potential target to forecast glioma patients’ tumor stage and prognosis, as well as a novel pharmacological target to enhance treatment outcomes and to find new cancer treatments.
